# Supplementary material for: The Streptococcus pyogenes hyaluronic acid capsule promotes experimental nasal and skin infection by preventing neutrophil-mediated clearance
Source: PLoS Pathog. 2022 Nov 30;18(11):e1011013. doi: 10.1371/journal.ppat.1011013 (PMC9744330; doi:10.1371/journal.ppat.1011013)
Supplement: S1 Fig — (PDF) [file ppat.1011013.s001.pdf]

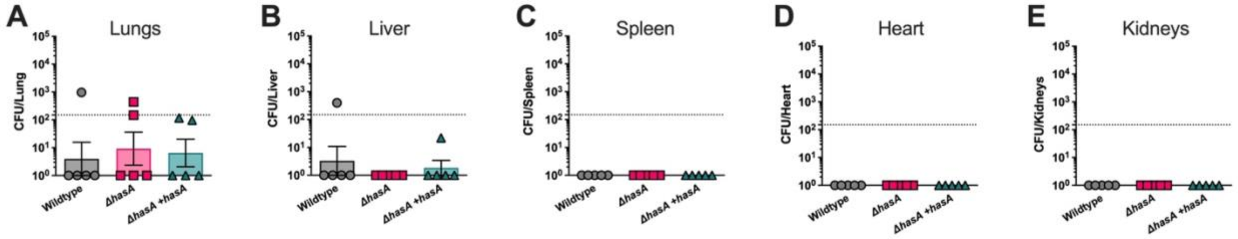

Fig S1. Deletion of the *hasA* gene in *S. pyogenes* MGAS8232 does not enhance bacterial dissemination in $B6_{HLA}$ mice. $B6_{HLA}$ mice were nasally challenged with $\sim 1\times10^8$ CFUs of wildtype *S. pyogenes* or Δ*hasA*. Mice were sacrificed 48 h later, and indicated organs were harvested, homogenized, and plated on TSA with 5% sheep blood agar to assess bacterial dissemination. Bacterial CFUs were measured in the **(A)** lungs, **(B)** liver, **(C)** spleen, **(D)** heart, and **(E)** kidneys. Data points represent CFUs of indicated organs from individual mice ($n \geq 4$ per group). Bars represent geometric mean ± SEM. Horizontal dotted line indicates theoretical limit of detection. Significance was determined by one-way ANOVA with Dunnett's multiple comparisons test, data not significant.
