# Supplementary material for: The Streptococcus pyogenes hyaluronic acid capsule promotes experimental nasal and skin infection by preventing neutrophil-mediated clearance
Source: PLoS Pathog. 2022 Nov 30;18(11):e1011013. doi: 10.1371/journal.ppat.1011013 (PMC9744330; doi:10.1371/journal.ppat.1011013)
Supplement: S2 Fig — (PDF) [file ppat.1011013.s002.pdf]

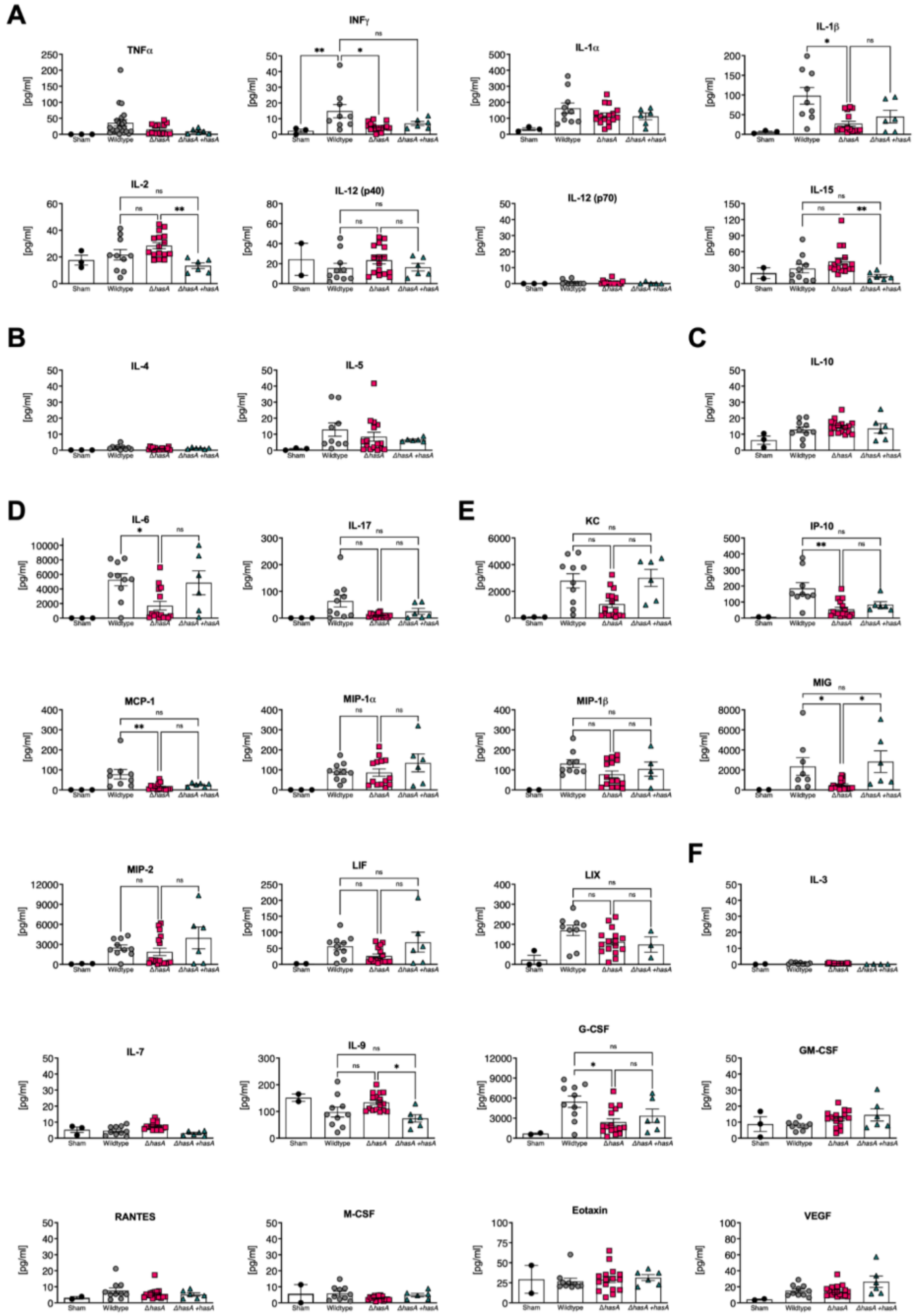

Fig S2. Cytokine response in nasal turbinates of $B6_{HLA}$ mice during streptococcal infection. Mice were inoculated with HBSS as sham control (black circles) or infected intranasally with ~1×10^8 CFUs of wildtype *S. pyogenes* MGAS8232 wildtype, Δ*hasA*, or Δ*hasA* + *hasA* strains. Mice were sacrificed 48 h post-infection and cNT homogenates were analyzed for multiple cytokines and chemokines (Th1-type [**A**]; Th2-type cytokines [**B**]; Treg cytokines [**C**]; Th17 cytokines [**D**]; chemokines [**E**]; or growth factors [**F**]). Data represents the mean ± SEM of cNT cytokine/chemokine concentrations (pg mL^{-1}) ($n \geq 3$ mice per group). Significance was determined by one-way ANOVA with Dunnett's multiple comparison test (* $P < 0.05$; ** $P < 0.01$; *** $P < 0.001$).
