# Supplementary material for: The Streptococcus pyogenes hyaluronic acid capsule promotes experimental nasal and skin infection by preventing neutrophil-mediated clearance
Source: PLoS Pathog. 2022 Nov 30;18(11):e1011013. doi: 10.1371/journal.ppat.1011013 (PMC9744330; doi:10.1371/journal.ppat.1011013)
Supplement: S1 Table — (PDF) [file ppat.1011013.s003.pdf]

Table S1. Primers in this study.

Plasmid	Primer Sequence (5′→ 3′)
Primers for *hasA* deletion constructs	
hasA up *Bam*HI For	CCC GGATCC TGTTTCCTTAATAAATAGTGTGAT
hasA up *Pst*I Rev	CCC CTGCAG TAAAGTTTTTTTAAAAATAGGCACAATTACACC
hasA down *Pst*I For	CCC CTGCAG AAAAAGGTCACTATTTTTAAATAATATATGCATCGAG
hasA down *Kpn*I Rev	CCC GGTACC TTCACAAGAAACAATAATTCGGCTTGG
Primers for sequencing and screening	
M13 For	GTAAAACGACGGCCAG
M13 Rev	GTCATAGCTGTTTCCTG
hasA Screen For	CTCTAAACTGCCTAACAGTTGGATAACACTC
hasA Screen Rev	GTGCAAATTTTTCTGCGTCTGCC
Primers for generating complements	
hasA Comp XhoI For	CCC CTCGAG GGGTGAAGTTTTTTTAATGGAAGCAC
hasA Comp SpeI Rev	CCC ACTAGTG AGGGAAGAATATCAACAATAGTGACTTCG

[1]Restriction sites (indicated in the primer name) are underlined in the primer sequence
