# Supplementary material for: The Streptococcus pyogenes hyaluronic acid capsule promotes experimental nasal and skin infection by preventing neutrophil-mediated clearance
Source: PLoS Pathog. 2022 Nov 30;18(11):e1011013. doi: 10.1371/journal.ppat.1011013 (PMC9744330; doi:10.1371/journal.ppat.1011013)
Supplement: S2 Table — (PDF) [file ppat.1011013.s004.pdf]

Table S2. SNPs identified through genome wide comparisons of wildtype *S. pyogenes* MGAS8232 and Δ*hasA* strains

Gene	Locus	Protein	Gene length (bp)	Codon number	Reference nucleotide	Competing nucleotide	Nucleotide in codon	Amino acid change
pstI[a]	spyM18_1384	AAL97979.1	1734	194	A	T	1	L > F
pstI[a]	spyM18_1384	AAL97979.1	1734	306	C	T	2	S > F

[a] cytosolic enzyme I in the phosphoenolpyruvate-protein phosphotransferase system
